# Enhanced Supercapacitor Performance Using Electropolymerization of Self-Doped Polyaniline on Carbon Film

**DOI:** 10.3390/nano8040214

**Published:** 2018-03-31

**Authors:** Po-Hsin Wang, Tzong-Liu Wang, Wen-Churng Lin, Hung-Yin Lin, Mei-Hwa Lee, Chien-Hsin Yang

**Affiliations:** 1 Department of Chemical and Materials Engineering, National University of Kaohsiung, Kaohsiung 81148, Taiwan; p5802341@gmail.com (P.-H.W.); tlwang@nuk.edu.tw (T.-L.W.); linhy@nuk.edu.tw (H.-Y.L.); 2 Department of Environmental Engineering, Kun Shan University, Tainan71070, Taiwan; linwc@mail.ksu.edu.tw; 3 Department of Materials Science and Engineering, I-Shou University, Kaohsiung 84001, Taiwan; mhlee@isu.edu.tw

**Keywords:** supercapacitor, self-doped polyaniline, carbon-nanoparticle film, electropolymerization, cyclic voltammetry

## Abstract

In this work, we electrochemically deposited self-doped polyanilines (SPANI) on the surface of carbon-nanoparticle (CNP) film, enhancing the superficial faradic reactions in supercapacitors and thus improving their performance. SPANI was electrodeposited on the CNP-film employing electropolymerization of aniline (AN) and o-aminobenzene sulfonic acid (SAN) comonomers in solution. Here, SAN acts in dual roles of a self-doped monomer while it also provides an acidic environment which is suitable for electropolymerization. The performance of SPANI−CNP-based supercapacitors significantly depends upon the mole ratio of AN/SAN. Supercapacitor performance was investigated by using cyclic voltammetry (CV), galvanostatic charge and discharge (GCD), and electrochemical impedance spectroscopy (EIS). The optimal performance of SPANI−CNP-based supercapacitor exists at AN/SAN ratio of 1.0, having the specific capacitance of 273.3 Fg^−1^ at the charging current density of 0.5 Ag^−1^.

## 1. Introduction

Energy storage systems are important nowadays because of the growing requirement for high power in charging and discharging, and thus the development of supercapacitors has attracted extensive attention recently. Supercapacitors have been categorized into electrochemical double layer capacitors (EDLCs) and pseudocapacitors according to their mechanism of charge storage [1]. EDLCs store charge employing electrical double-layer electrostatic force in an electrochemical double layer (Helmholtz Layer) [2]. The main materials used in EDLC are carbon-based materials, such as graphene, carbon nanotubes, carbon black, etc. In pseudocapacitors, charge is stored via fast and reversible faradic reaction, which is based on electroactive materials with several oxidation and reduction states (e.g., metal oxides and conducting polymer). In general, the capacitance of pseudocapacitors are higher than EDLCs [3,4]. On the other hand, EDLCs use very stable carbons to increase voltage (V) [5,6].

Conducting polymers, such as polyaniline (PANI) which is a potential material for supercapacitors due to its excellent electrochemical activity, ease of synthesis, environmental stability, and high specific capacitance [7], have been used for supercapacitors. Based on previous studies, the nanosized materials can improve supercapacitor electrodes by increasing the area of the electrode–electrolyte interface and decreasing the cation diffusion length within the active material [8,9,10]. Electrically conductive nanosized PANI is combined with porous carbon, carbon nanotubes, or graphene as a nanostructured template that not only improves the charge/discharge cycle of PANI but also promotes the specific capacitance to 233–1220 Fg^−1^ [8,11,12,13,14].

Self-doped polyanilines (SPANI) nanofibers have been synthesized by the molecular self-assembly process using aniline (AN) and o-aminobenzenesulphonic acid (SAN) without extra addition of inorganic acids. SAN acted as a self-doping monomer, surfactant, and template for PANI nanofibers due to its hydrophilic group (–SO_3_H). Because SAN acted as a self-doping monomer and was simultaneously linked in the polymer chains, the SAN didn’t need removal after polymerization. We tried to employ this method devoid of extra addition of inorganic acids, which is significantly different from the alternative template methods and external doping systems [15,16]. 

In this work, SPANIs with different AN/SAN ratios were deposited on the surface of carbon nanoparticle (CNP) film by electropolymerization forming SPANI−CNP composite electrodes. The SPANI−CNP composites are characterized by their morphologies, structure, and composition through examination by scanning electron microscopy (SEM), X-ray diffraction (XRD), Fourier transfer infrared spectrometer (FTIR), and X-ray photoelectron spectroscopy (XPS). The effects of AN/SAN ratio in SPANI−CNP composite electrodes on the performance of supercapacitors was investigated in three-electrode type and symmetric cells, respectively. The supercapacitor performance of the SPANI−CNP composite electrodes with different AN/SAN ratios was studied by cyclic voltammetry (CV), galvanostatic charge–discharge (GCD), and electrochemical impedance spectroscopy (EIS). The assembled SPANI−CNP-based supercapacitors demonstrate the optimal performance, having SPANI film at the AN/SAN ratio of 1.0 which provides channels for rapid transportation of electrolyte ions coupled with extra pseudocapacitive capabililty.

## 2. Experimental Section

### 2.1. Materials

Aniline (Aldrich, Milwaukee, WI, USA) was distilled under reduced pressure. o-Aminobenzenesulfonic acid (SAN, Fluka, Buchs, Switzerland) was recrystallized two times in distilled water. Carbon nanoparticles (~13 nm, Colour Black FW200, UniRegion Bio-Tech, Taip, Taiwan), poly(vinylidene fluoride) (PVDF, Solf® PVDF 6020, Solvay, Bruxelles, Belgium), 1-methyl-2-pyrrolidone (NMP, Riedel-de Haën, Seelze, Germany), graphite foil (Hongye Vacuum Technology Co., Ltd., Xinzhu, Taiwan), and sulfuric acid (Fluka, Buchs, Switzerland) were used as received.

### 2.2. Preparation of CNP Substrates

The carbon nanoparticle (CNP) electrodes were prepared by the following procedures. Graphite foil as the current collector was polished by sandpaper and washed by 0.05 M H_2_SO_4_, then dried in a vacuum oven at 120 °C for 2 h. Then, carbon nanoparticles and PVDF were mixed in a mass ratio of 9:1 by Agate mortar and dispersed in the NMP solvent, grinding until the paste was homogeneous. The paste was dropped onto a graphite foil (2 cm × 1 cm) and dried at 70 °C for 16 h in a vacuum oven. The loading mass of CNP was about 0.5 mg∙cm^−2^.

### 2.3. Preparation of SPANI−CNP Composite Electrodes

Monomers of AN and SAN was dissolved in 50 mL of distilled water with total concentration of 0.1 M at various mole ratio of AN/SAN from 0.5 to 4. SPANI was electrodeposited on CNP substrates forming SPANI−CNP composite electrodes using cyclic voltammetry in a three-electrode cell equipped with Pt counter electrode and reference electrode of Ag/AgCl. The potentials swept from −0.2 to 0.8 V (vs. Ag/AgCl) scanning ca. 40 cycles at the scan rate of 25 mV∙s^−1^. The deposited SPANI−CNP composite electrodes were dried in vacuum oven at 60 °C for 16 h.

### 2.4. Characterization Techniques

The surface morphology of the SPANI−CNP composites was identified by field-emission scanning electron microscopy (FE-SEM, JSM-6700F, JEOL Ltd., Tokyo, Japan). Infrared spectra were recorded on an FTIR spectrometer (Agilent Technologies, Cary 630, Santa Clara, CA, USA) to check the functional groups on polymer films. The structural and chemical composition were determined by X-ray photoelectron spectroscopy (XPS) using a Theta Probe AR-XPS (Theta 300 Version, Thermo Fisher Scientific, Paisley, UK) which scanned the surface of the sample with a monochromatic X-Ray beam source of 1486.6 eV (aluminum anode) and 15 kV.

### 2.5. Electrochemical Characterization

Electrochemical characterization of SPANI−CNP composite electrodes in the respective three-electrode cells and symmetric cells were analyzed using cyclic voltammetry (CV), galvanostatic charge and discharge (GCD), and electrochemical impedance spectroscopy (EIS) on an AUTOLAB PGSTAT302N electrochemical work station (Metrohm Autolab, Utrecht, Netherland). 1 N H_2_SO_4_ was quoted as the electrolyte solution_._ Three-electrode cells were equipped with the SPANI−CNP working electrode, platinum plate counter electrode, and reference electrode of Ag/AgCl electrode, respectively. The cyclic voltammetry (CV) data were recorded with scan rates of 5 and 10 mV∙s^−1^ over the potential window of −0.2–0.8 V in a three-electrode cell. In the symmetric cell, the CV data were obtained at various scan rates of 10, 25, 50, 75, 100, and 125 mV∙s^−1^ over the potential window of 0–0.8 V. The galvanostatic charge–discharge (GCD) curves were obtained over the potential range of 0–0.6 V at various current densities of 0.5, 1, 1.5, 2, 2.5, 5, and 10 A·g^−1^ in symmetric devices. A current density of 1 and 5 A·g^−1^ was used in three-electrode experimental cells over the potential range of 0–0.8 V (vs. Ag/AgCl). The electrochemical impedance spectroscopy (EIS) measurements were carried out between the frequency range of 0.1 Hz to 100 kHz at an amplitude of 10 mV.

## 3. Results and Discussion

### 3.1. Cyclic Voltammograms of SPANI Electropolymerization on CNP Substrate

**Figure 1:**
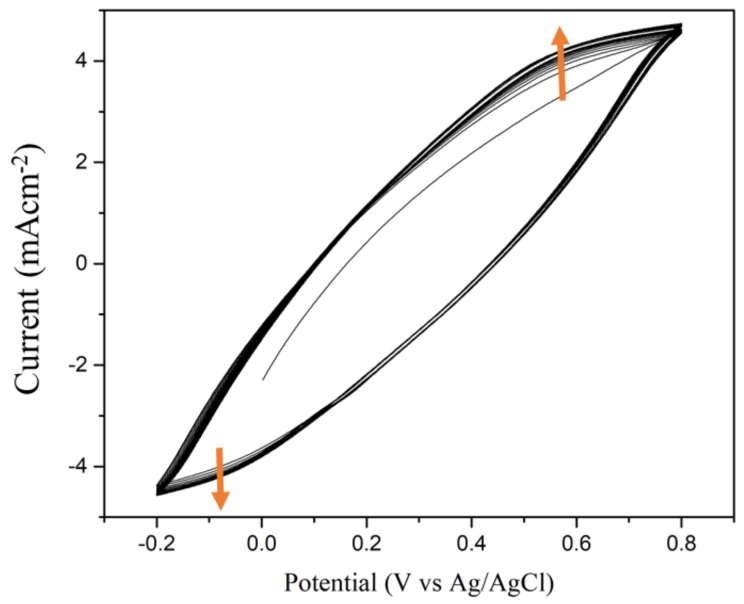
Cyclic voltammograms of self-doped polyanilines (SPANI) deposited on a carbon nanoparticle (CNP) substrate using electropolymerization with AN/SAN mole ratio of 1.0 (pH = 5.0) at a scan rate of 25 mV∙s^−1^. Yellow arrows represent the direction of current increase.

Electropolymerization of polyaniline needs attachment and chain extension between active intermediates of diradical dications [17] generated from the monomers and propagating oligomer chains. The diradical dication is an energetic electrophile, so polymerization is generally performed in the presence of a strong acid (e.g., 1 M HCl, pH = −0.15) to deposit polyaniline on the electrode. Because the SAN molecule has a sulfonic acid group (–SO_3_H) in the ortho-position on the benzene ring, SAN molecules can generate the diradical dications (the bipolaronic form of pernigraniline, presents peak at ~800 mV in pH = −0.15) [18] through self-doping amino groups (–NH_2_ on AN and SAN molecules). This species is an energetic electrophile, extracting an electron from AN and SAN and becoming a radical cation which bonds with another radical. The increase in the intensity of peak at ~250 mV (in pH = −0.15) [18] indicates that these radical cations undergo bonding to form the polymer deposited on the substrate. Alternatively, we designed that the sulfonate-containing SAN mixed with AN as a comonomer to make a weakly acidic aqueous solution (pH = 5.0) without adding inorganic acid. Figure 1 shows the cyclic voltammogramms (CVs) of AN-SAN electropolymerization on a CNP substrate. In this weak acidic solution, the current still gradually increases with increasing the cycling number of electropolymerization, due to the increase in deposition of the conductive polymer. As above, when adding 1 M HCl to AN-SAN comonomers (pH= −0.15), the corresponding CVs of electropolymerization presented two distinct redox couples around at ~250 mV and ~800 mV [18]. But in this work (pH = 5.0), the above two peaks of diradical dications and radical cation are merged together to become indistinguishable in this weakly acidic environment.

### 3.2. Material Analysis

**Figure 2:**
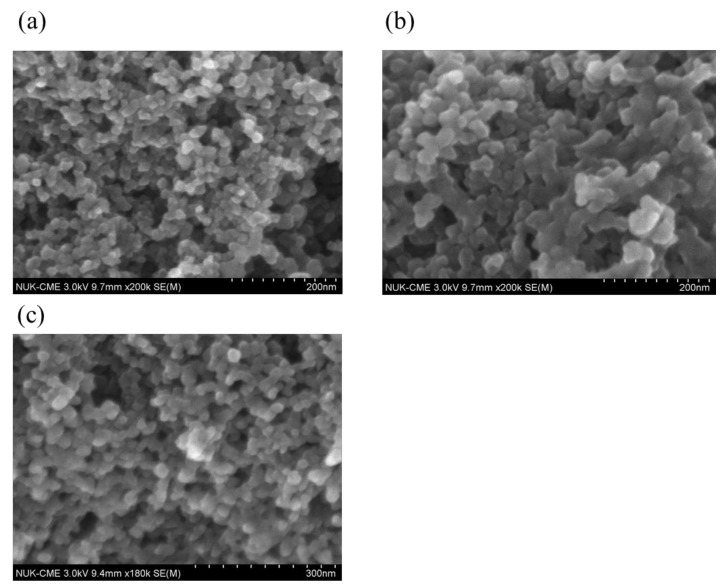
SEM images of (**a**) carbon nanoparticles; (**b**) SPANI−CNP composites with an AN/SAN ratio of 1.0; and (**c**) SPANI−CNP composites with an AN/SAN ratio of 4.0.

Figure 2 shows the morphology of the CNP and SPANI−CNP composites. The CNP nanoparticles with the size of 20 nm can be seen in Figure 2a. The SPANI was assembled onto CNP film, thus enlarging the particle size to ~50 nm as shown in Figure 2a,b. As compared the size of CNP nanoparticles, the increase of particle size of SPANI−CNP composites arises from assembling the SPANI deposition onto CNP particles having SPANI polymers acting as a wrapping layer.

**Figure 3:**
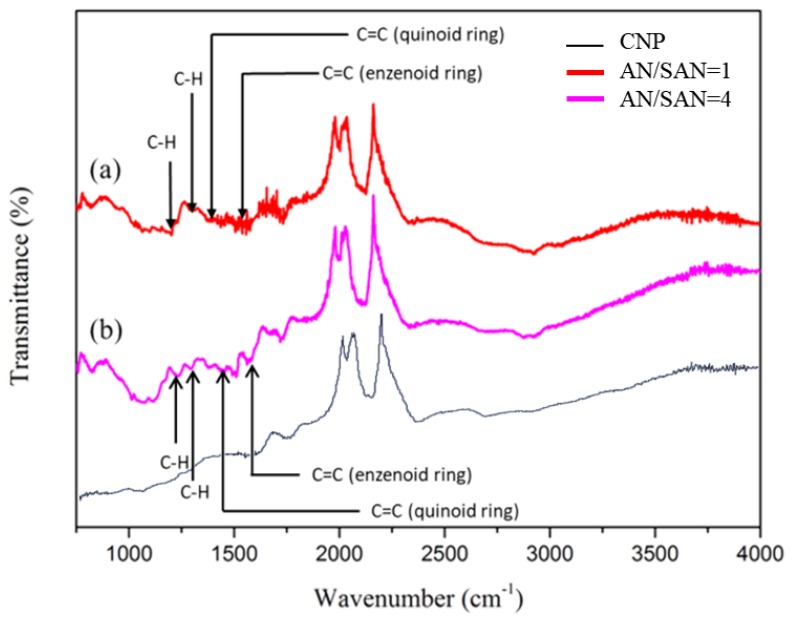
FTIR spectra of SPANI−CNP composites with AN/SAN ratio of (a) 1.0 and (b) 4.0.

Figure 3 shows FTIR spectra of SPANI−CNP composites with AN/SAN ratio of 1 and 4, respectively. There are similar FTIR results in the two SPANI−CNP composites. The characteristic peaks of 1375 cm^−1^ and 1513 cm^−1^ are assigned to C=C stretching vibration of the quinoid ring and benzenoid ring, respectively. The bands at 1204 cm^−1^ and 1304 cm^−1^ are assigned to C–H stretching vibration with aromatic conjugation [16].

**Figure 4:**
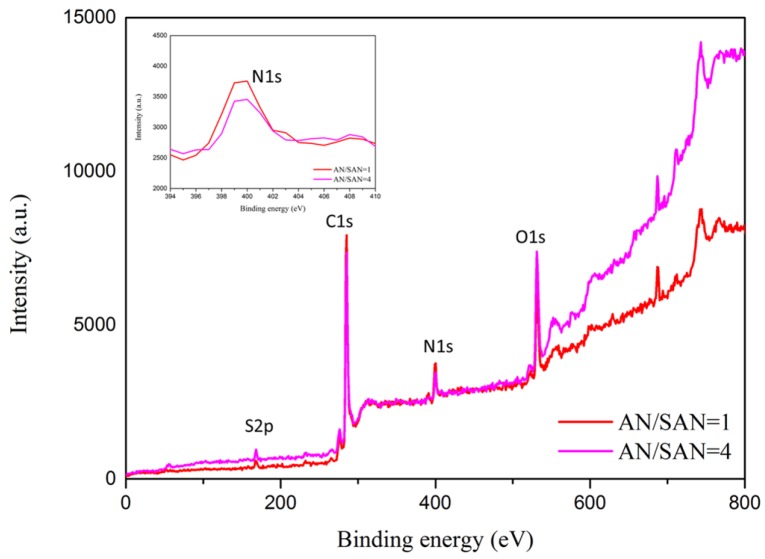
XPS spectra of SPANI−CNP composites with different AN/SAN ratios.

**Figure 5:**
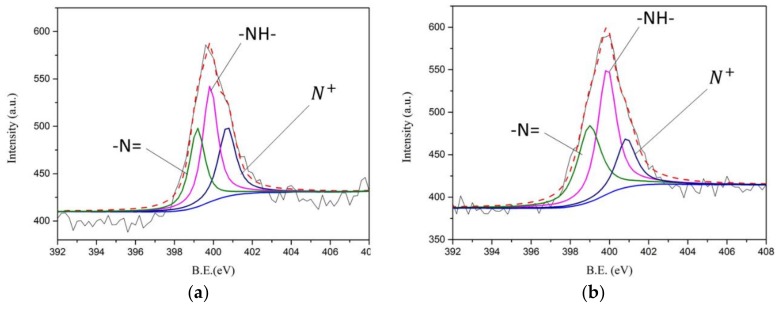
N_1s_ XPS core-level spectra of the SPANI−CNP with AN/SAN mole ratio of (**a**) 1 and (**b**) 4.

In Figure 4, the atomic composition of the SPANI−CNP composites with different AN/SAN ratio is studied using X-ray photon spectroscopy (XPS), exhibiting the existence of C, N, O, and S elements. The N_1s_ deconvolution of the SPANI−CNP composites with AN/SAN ratio of 1 and 4 are shown in Figure 5a,b. The binding energies of 399.2, 399.8, and 400.8 eV correspond to the imine (–N=), amine (–NH–), and polaron species (N^+^), respectively [12]. The formation of polaron species is contributed by the nitrogen in the vicinity of H^+^ cations when the polymers were self-doped with –SO_3_H groups on the polymer chains. The formation of imine sites is due to the strong hydrogen bonding of –NH to oxygen atoms. Table 1 lists the results of N_1s_ deconvolution and doping degree ([N^+^]/[N]), revealing the doping degree of SPANI−CNP composites with AN/SAN ratio of 1 higher than that with AN/SAN ratio of 4. This is because the former has a higher content of SAN compared to the latter. Based on previous report, the electrical conductivity of SPANI is proportional to the doping degree [16]. As a result, it is conceivable that the conductivity of SPANI−CNP composites with AN/SAN ratio of 1 is higher than that with AN/SAN ratio of 4. 

### 3.3. Electrochemical Studies

**Figure 6:**
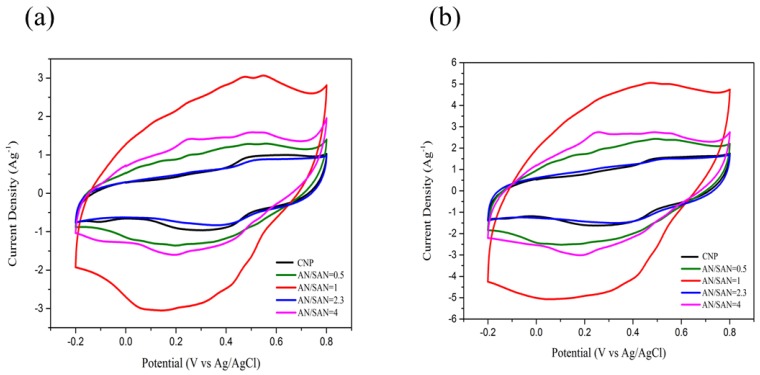
CV curves of SPANI−CNP composite electrodes with AN/SAN ratio at scan rates of (**a**) 5 mV∙s^−1^.and (**b**) 10 mV∙s^−1^.

Electrochemical techniques of cyclic voltammetry (CV), electrochemical impedance spectroscopy (EIS), and galvanostatic charge–discharge were employed to study the performance of CNP and SPANI−CNP composite-based electrodes in a three-electrode cell and a symmetric cell, respectively. The specific capacitance value (*C*_s_) of CNP- and SPANI−CNP composite-based electrodes can be calculated from the CV curves according to equation [8]:

(1) $$C_{s}=\frac{\int Idv}{vm\Delta V}$$

where *m* is the mass of the electroactive material (g), *v* is the sweep rate (V·s^−1^), ∆*V* is the potential range (V), and the integrated area under the CV curve *I* is the response current (A). According to Equation (1), the integrated area of CV curve is proportional to specific capacitance of electrodes, so the specific capacitance of the CNP electrode and SPANI−CNP composite electrodes with AN/SAN ratios of 0.5, 1.0, 2.3, and 4.0 were compared. Figure 6a,b shows the cyclic voltammograms (CVs) in a three-electrode cell at sweep rate of 5 and 10 mV∙s^−1^ in 1 N H_2_SO_4_ solution. In these CVs, the peaks of oxidation and reduction peaks were observed from the characteristics of SPANI. Figure 6a,b also indicate that the SPANI−CNP composite electrodes showed a higher specific capacitance and electrochemical performance as compared CNP electrode. Moreover, the SPANI−CNP composite electrode with AN/SAN ratio of 1.0 possessed the highest specific capacitance than other AN/SAN compositions. These could be attributed to higher electrical conductivity of SPANI and its Faradic contribution.

Faradic contribution provided pseudocapacitance which was caused by reversible redox transition involving the exchange of protons (or cations) in the electrolyte (H_2_SO_4_) explained as follows.

SPANI + H^+^ + e^−^ ↔ SPANI-H (2)

where SPANI:

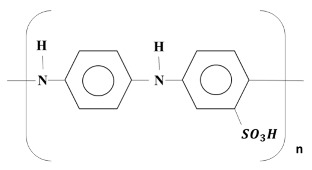

where H^+^ denotes the protons in the electrolyte (H_2_SO_4_). Protons come from the sulfonic acids of SAN units in polymer chains. This implies that the formation of more complete polaronic structure of SPANI chains at this case. The above equation suggests that both protons are involved in the redox process. Here, SPANI promotes faster charge–discharge rates through the Faradaic reaction arising from doping–dedoping of the self-doped groups of sulfonic acid in the SAN units. These results further indicate that charge stored at the surface of the SPANI from the pseudocapacitance storage mechanism is an important factor in achieving high gravimetric energy density values.

**Figure 7:**
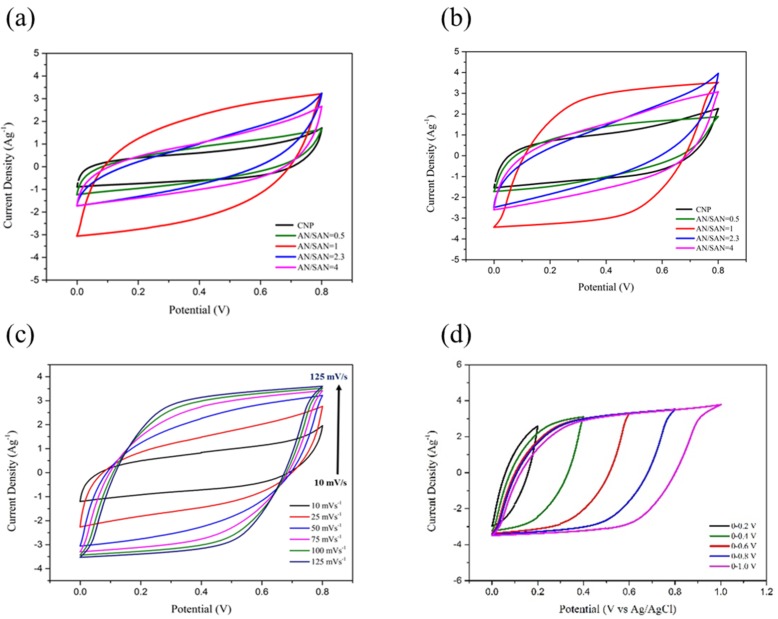
CV curves of SPANI−CNP composites with different AN/SAN ratios and CNP electrodes at scan rates of (**a**) 50 mV∙s^−1^ and (**b**) 100 mV∙s^−1^; (**c**) CV curves of SPANI−CNP composites with different AN/SAN ratios and CNP electrodes at scan rates of 100 mV∙s^−1^; (**d**) CV curves of SPANI−CNP composite with AN/SAN ratio of 1.0 in the voltage-window range of 0–1.0 V.

Figure 7a,b shows the CVs in a symmetric cell based on SPANI−CNP composite and CNP electrodes at sweep rate of 75 and 100 mV∙s^−1^ in 1 N H_2_SO_4_ solution. The symmetric cell based on the SPANI−CNP composite electrode with an AN/SAN ratio of 1.0 yielded the largest area in the CVs. Figure 7c shows the CVs in a symmetric cell based on SPANI−CNP composite electrode supercapacitor with an AN/SAN ratio of 1.0 at scan rates of 10, 25, 50, 75, 100, and 125 mV∙s^−1^. These CVs have an almost rectangular shape from 10 mV∙s^−1^ to 125 mV∙s^−1^, indicating fast transport of electrolyte ions and excellent capability. This represents that the charge transport on the surface of this electrode is highly efficient [9]. Figure 7d shows the CVs of the SPANI−CNP composite electrode with an AN/SAN ratio of 1.0 at various voltage windows, indicating that the shape of these CV curves remains almost identical in the potential range of 0.2 V to 1.0 V. This means that the SPANI−CNP composite electrode with an AN/SAN ratio of 1.0 has excellent capacitive characteristics and reversibility. The CVs exhibit a tail at the upper limit potential of 1.0 V, indicating the evolution of oxygen [9].

### 3.4. Electrochemical Impedance Spectroscopy

**Figure 8:**
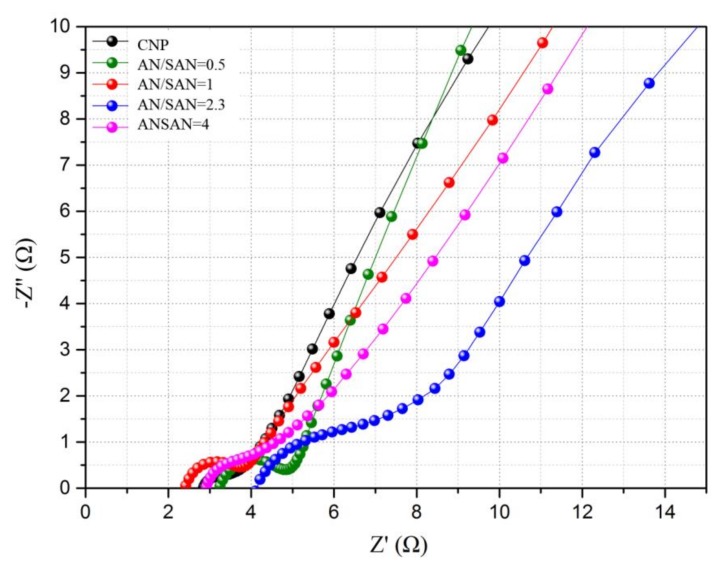
Nyquist plot of CNP and SPANI−CNP composite electrodes.

The interface behavior of the electrode materials in supercapacitors is usually studied using EIS analysis [15]. The EIS experiment was performed in the frequency range of 0.1 to 100 kHz at an amplitude of 10 mV. In Figure 8, the Nyquist plot of CNP and SPANI−CNP composite electrodes showed a semicircular arc in the high-frequency range and a straight line in the low-frequency range. The arc of the high-frequency range indicates the charge transfer limiting process arising from the double-layer capacitance coupled with the charge transfer resistance (*R*_ct_) at the contact interface between the electrode and electrolyte solution [10]. Figure 8 shows that the SPANI−CNP composite electrode with an AN/SAN ratio of 1.0 has the smallest diameter of the semicircular arc at the high-frequency region. The values of *R*_ct_ for the CNP and SPANI−CNP composite electrodes with AN/SAN ratios of 0.5, 1.0, 2.3, and 4.0 are 1.0, 1.89, 1.63, 4.0, and 1.97, respectively (refer to Table 2). The SPANI−CNP composite electrode with an AN/SAN ratio of 1.0 exhibited the lowest *R*_ct_ value among these SPANI−CNP composite electrodes, indicating that the SPANI composite with AN/SAN ratio of 1 has a relatively lower R_ct_ and better capacitive behavior with small diffusion resistance [10]. On the other hand, the interception on the x-axis at the high-frequency region was used to estimate the equivalent series resistance (ESR). The ESR values can be obtained for the CNP and SPANI−CNP composite electrodes with AN/SAN ratios of 4.0, 2.3, 1.0, and 0.5 are 2.83, 2.9, 4.1, 2.39, and 3.23 Ω, respectively (refer to Table 2). The SPANI−CNP composite electrode with an AN/SAN ratio of 1.0 exhibits the lowest ESR value. This situation could be attributed to the highest conductivity of SPANI with an AN/SAN ratio of 1.0 [16] and the incorporation of SPANI into the composite structure enhancing efficient transport of electrolyte ions to the surface of the composite electrodes. The SPANI polymer chains facilitate the attachment of H^+^ ions for electrical neutrality. In the low-frequency region, the straight line corresponds to the Warburg resistance, which is caused by frequency dependence of ion diffusion and transport among the electrolyte to the electrode surfaces [10]. The almost vertical line of the SPANI−CNP composite electrode with an AN/SAN ratio of 1.0 in the low-frequency range indicates fast ion diffusion in the electrolyte and adsorption/desorption onto the electrode surface.

### 3.5. Galvanostatic Charge−Discharge

**Figure 9:**
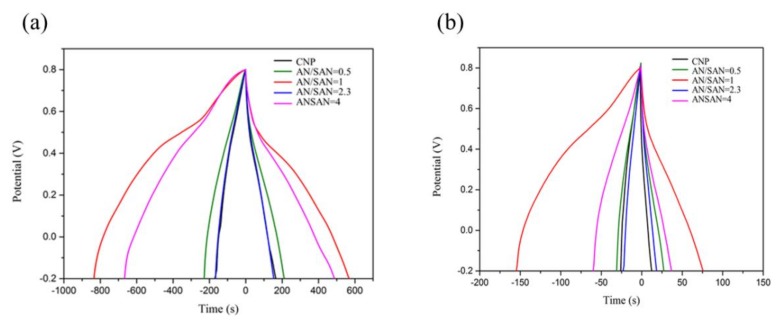
Galvanostatic charge and discharge curve of CNP and SPANI−CNP composite electrodes with AN/SAN ratio at current density of (**a**) 1 A·g^−1^ and (**b**) 5 A·g^−1^ in 1 N H_2_SO_4_.

The specific capacitance of three-electrode and tow-electrode systems can be calculated from the galvanostatic charge–discharge curve at particular current densities by using Equations (3) and (4) [12], respectively:

(3) $$C_{s}=\frac{I\Delta t}{m\Delta V}$$

(4) $$C_{s}=\frac{4I\Delta t}{m\Delta V}$$

where *I* is the discharge current (mA), *m* is the mass of total electroactive material (g), ∆*V* is the potential range of charge and discharge, and ∆*t* is the discharge time (t). Figure 9 shows the galvanostatic charge and discharge curves of SPANI−CNP electrodes in a three-electrode system, where CNP and SPANI−CNP composites, platinum, and Ag/AgCl were used as the working electrode, the counter electrode, and the reference electrode, respectively, at a constant current density of 1 and 5 A·g^−1^ in 1 N H_2_SO_4_ electrolyte solution within a potential window of 0–0.8 V. The charge and discharge curves on CNP electrodes have a symmetric triangular shape, indicating the behavior of an electrical double-layer capacitor (EDLC) [11]. But, a deviation from a straight line was shown in the galvanostatic charge–discharge curves of the SPANI−CNP composite electrodes, indicating pseudocapacitive behavior [14]. The SPANI−CNP composite electrode with an AN/SAN ratio of 1.0 exhibited the highest specific capacitance in these devices.

**Figure 10:**
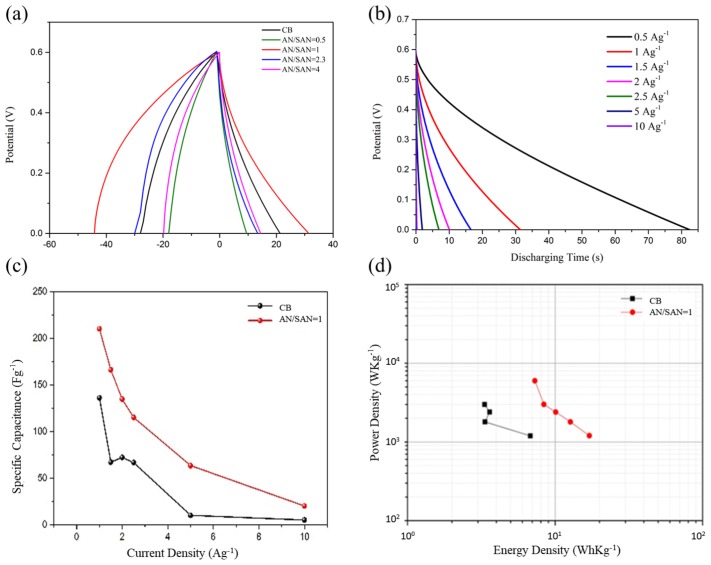
(**a**) Galvanostatic charge and discharge curves of CNP and SPANI−CNP composite electrodes with AN/SAN ratio at current density of 1 A·g^−1^; (**b**) Discharging times of SPANI−CNP with AN/SAN = 1 composite at different current density; (**c**) Rate capability of CNP and SPANI−CNP composite electrodes; (**d**) Ragone Plot of CNP and SPANI−CNP-based supercapacitors.

Figure 10a shows the galvanostatic charge and discharge curves of SPANI−CNP electrodes in a symmetric cell at a constant current density of 1 A·g^−1^ in 1 N H_2_SO_4_ electrolyte solution within a potential window of 0–0.6 V. The specific capacitances of the CNP and the SPANI−CNP-based supercapacitors in the symmetric cells with AN/SAN ratios of 0.5, 1.0, 2.3, and 4.0 are 68, 53.3, 213.3, 102.6 and 96.6 F·g^−1^, respectively, at current density of 1 A·g^−1^. The SPANI−CNP composite with an AN/SAN ratio of 1.0 had the highest specific capacitance among these symmetric cells. Figure 10b shows the discharging rate of SPANI−CNP composite with AN/SAN ratio of 1 at different current density, revealing that the discharging time increased with decreasing of the discharging current density. Figure 10c demonstrates the rate capability of CNP and SPANI−CNP-based capacitors, indicating that the specific capacitance decreases with increasing current density and the specific capacitance of the SPANI−CNP-based capacitor is higher than that of CNP-based capacitor.

Specific energy density (E, Wh∙kg^−1^) and specific power density (P, W∙kg^−1^) can be calculated using the following equations [14]:

(5) $$E=\frac{1}{2}CsV^{2}$$

(6) $$P=\frac{E}{t}$$

where *C*_s_ is the specific capacitance of the supercapacitors, *V* is the voltage range during the discharge process in galvanostatic charge and discharge curves, and t is the discharge time. Figure 10d shows the Ragone plot, indicating improvement in energy density (14.1 Wh/Kg) of SPANI−CNP-based capacitor with AN/SAN ratio of 1 as compared that (6.8 Wh/Kg) of CNP-based capacitor. This improvement in energy density can be attributed to the enhancement of effective cation diffusion in the SPANI−CNP composites.

**Figure 11:**
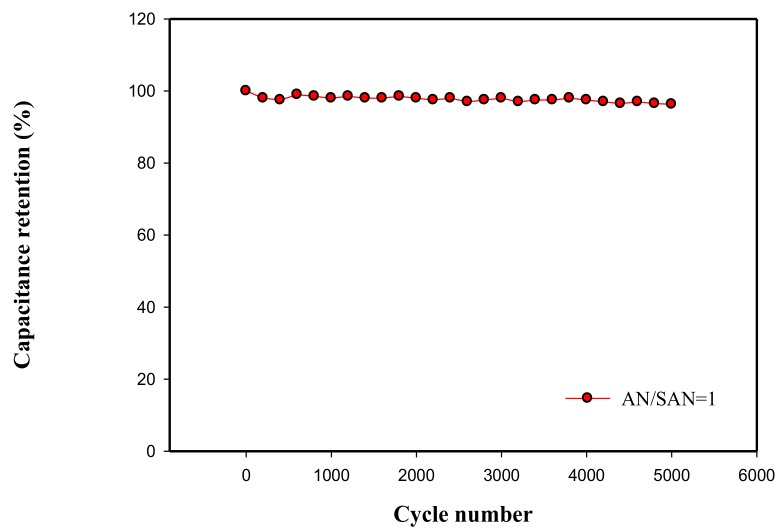
Cycling stability of SPANI−CNP-based supercapacitor at current density of 2 A·g^−1^.

A SPANI−CNP-based supercapacitor with AN/SAN ratio of 1 was employed to test for the cycle stability though galvanostatic charge and discharge in current density of 2 A·g^−1^ for 5000 cycles. Figure 11 shows 96.1% retention in capacitance after 5000 cycles, indicating excellent cycle stability in this supercapacitor.

## 4. Conclusions

We electrodeposited self-doped polyaniline (SPANI) onto carbon nanoparticle (CNP) film to form a SPANI−CNP composite by electropolymerization and improved the capacitive performance of the CNP electrode through increasing its conductivity and psuedocapacitive characteristic. SPANI with AN/SAN ratio of 1 exhibited the best performance among these AN/SAN ratios. In galvanostatic charge and discharge, the SPANI−CNP composite with AN/SAN ratio of 1 exhibited maximum specific capacitance of 273.3 F·g^−1^ compared to 133.3 F·g^−1^ in the CNP electrode, the specific energy density and power density of SPANI−CNP composite with AN/SAN ratio of 1 were 7.3 Wh∙Kg^−1^ and 5996.8 W∙Kg^−1^ compared to 3.3 Wh∙Kg^−1^ and 2997 W∙Kg^−1^ for the CNP electrode. Moreover, the capacitance retention was 96.3% after 5000 cycles in galvanostatic charge and discharge. Our results have shown that the SAN can replace other external doping and have the same supercapacitive performance as polyaniline/carbon composites. The SPANI−CNP composite with AN/SAN ratio of 1 had a superior supercapacitive performance due to its Faradic contribution and its efficient charge-transfer and ion transport between electrolyte and electrode. These results demonstrated that the SPANI−CNP composites have practical potential for electrochemically stable supercapacitors.

## Figures and Tables

**Table 1:** Relative ratio (%) of different nitrogen components in SPANI–CB composites from N_1s_ XPS spectra.

AN/SAN	–N = (eV/%)	–NH– (eV/%)	$N^{+}$ (eV/%)
1	399.2 (28.5%)	399.8 (39%)	400.8 (32.4%)
4	399 (31.6%)	399.8 (42.1%)	401 (26.2%)

**Table 2:** ESR and *R*_ct_ values of CNP and SPANI−CNP composite electrodes.

Sample	ESR (Ω)	R_ct_ (Ω)
CNP	2.83	1.0
AN/SAN = 0.5	3.23	1.89
AN/SAN = 1	2.39	1.63
AN/SAN = 2.3	4.1	4.0
AN/SAN = 4	2.9	1.97
